# Electric-Field Nanobubble-Enhanced Progress in Anaerobic Digestion Unit Operations: Biogas Upgrading and Up- and Down-Stream Water and Sludge Treatment Operations

**DOI:** 10.3390/nano15130968

**Published:** 2025-06-22

**Authors:** Niall J. English

**Affiliations:** School of Chemical & Bioprocess Engineering, University College Dublin, Belfield, D04 V1W8 Dublin, Ireland; niall.english@ucd.ie

**Keywords:** nanobubbles, anaerobic digestion, water treatment

## Abstract

An integrated approach is sorely needed to treat biogas emanating from anaerobic digesters (AD) which is cost-effective, in terms of upgrade/purification to ~95–98% methane needed for pipeline injection. This is a very pressing environmental and waste-management problem. At present, biogas water-/solvent-washing operations require significant capital investment, with high operational and maintenance costs. In the present study, we deployed a facile and efficient novel nanobubble-formation approach using applied electric fields to boost biogas-enrichment operations: we achieve substantial methane enrichment via selective CO_2_ and H_2_S take-up in water in the form of nanobubbles. This enables an integrated waste-processing vision using cutting-edge engineering-science advances, and making anaerobic digestion a circular-economic and practical reality, that can be deployed at scale—initially developing at the small scale—and points the way for low-energy CO_2_ capture in the form of nanobubbles by dint of the electric-field approach. In addition, we carried out nanobubble generation using various gases for water treatment for both up- and down-stream sludge-containing (waste)water, achieving meaningful operational successes in AD operations and organic-fertiliser production, respectively.

## 1. Introduction

Anaerobic digestion (AD) is the bacterial conversion of an organic feedstock by microorganisms in the absence of oxygen into biogas and digestate. In order to achieve its potential as a renewable fuel the biomethane generated from AD must be purified to “fossil grid” specification with a purification of >98%, e.g., for EN-16723 grid-injection standards [1]. The primary constituents of biogas from AD consists of methane 50–70 v%, CO_2_ 30–50%, and H_2_S < 1%). The contaminants are very corrosive, especially H_2_S, and also inhibit combustion; therefore, its maximum available energy value as a ‘renewable national-grid gas’ or ‘renewable transport gas’ can only be achieved by purifying methane to 98 v%. Biomethane purification has been achieved by various competing technologies, such as pressure-swing adsorption (PSA), cryogenic separation, chemical washing (water, amines, alkaline solutions) and membrane technology; unfortunately, these all have high capital and operating costs (CAPX & OPEX). These are optimal at greater than 500 m^3^/h (STP) of biogas, escalating significantly at throughputs of less than 200 m^3^/h.

The trend throughout Europe is for smaller (20–200 m^3^/h) biomethane-purification capacities. It will enable a very wide range of potential small anaerobic-digester users, such as dairy farmers with 50–80 cows, and late-cut silage and beet, to become self-sufficient in producing enough bio-methane to support processes (home/dairying operations) and transport (car/tractor, etc.), as well as AD operations at wastewater-treatment plants.

Small-scale AD has enormous potential globally to conveniently supply renewable energy locally (secure from global energy supply volatility). It is only in Scandinavia and New Zealand where sophisticated, but very capital- and labour-intensive, AD-aggregation models are used, and where they make significant contributions to national-energy inventories. Moreover, the initial goal of this project is to develop this technology in the small-scale market and demonstrate that the technology is scalable linearly; from this initial experience, it has the potential to work equally well for larger-throughput facilities, generating substantial CAPEX and OPEX savings in wastewater treatment.

NBs are gas bubbles that are defined by their diameter, which is typically in the nanometre range. This very small size gives NBs characteristics that are very different from larger micron-sized bubbles. NBs typically exhibit greater metastability when compared to their micron-sized counterparts, and they exhibit several unique physical and mechanical characteristics. These include an enhanced solubility of a gas in water, an extremely high surface area/volume ratio, reduced or virtually eliminated buoyancy, greater zeta potentials, and the generation of free radicals [2,3,4], sometimes due to electrostatic effects from dipolar alignment [5]. In contrast, micron-sized bubbles decrease in size and eventually disappear underwater because of the rapid dissolution of the interior gas. This patented, innovative nanobubble (NB) technology has potential to reduce at least several-fold the CAPEX and OPEX of AD [1], and it is for small-scale operations where the NB technology offers a competitive and key advantage, despite using NB-enhanced water-washing operations for biogas upgrade as an inherently scalable technology. The bench-scale results for gas-in-liquid accommodation have shown that several-fold increases for gases, above achievable according to Henry’s Law solubility level, have been realised by using this new technology; the NBs in the liquid are highly metastable.

Surveying some advances of nanobubble applications in the important wastewater field, by using mechanisms like collisions, attachment, and detachment [6] in the flotation process, NB-enhanced floatation operations in seawater desalination have demonstrated promise in more recent studies on NBs in water treatment and flotation. Optimising NB-mediated flotation phenomena in mineral recovery and flotation kinetics in general has been investigated in terms of the best way to generate and detect NBs (e.g., optimal surfactant deployment strategy), such as electrolysis, hydrodynamic cavitation, and ultrasonication) [7]. Due to their increased mass-transfer rates and collision efficiency, among other benefits, micro-nanobubbles have also recently been more thoroughly studied and appreciated, making them particularly promising for water treatment applications like flotation and disinfection [8]. These more recent studies of NBs and their potential to enhance flotation and water treatment processes provide more economical and efficient alternatives to the current state-of-the-art aeration bubbling methods [6,7,8].

A key application area where nanobubble generation is expected to have a large impact is differential CO_2_-stripping from biogas, or biogas ‘upgrade’—originating from anaerobic digestion (AD)—with the European biogas market set to reach USD 10–11 billion by 2026 (*vide infra*) [1,9].

In terms of previous exploration for the role of nanobubble sin improving the efficiency and performance of AD unit operations, let us consider some foundational previous work. Considering methanogenesis and hydrolysis per se, it has been found that nanobubbles improve these rate-limiting AD processes: NBs boost substrate accessibility and enzyme activity because of their small size and large surface area per unit volume [10]. In an AD context, nanobubbles can also serve to boost facultative bacteria, enhance the electron transport system, and lower the level of sulpides and volatile fatty acids [10,11]. Increased microbial activity in AD processes has also been found by NBs, with enhanced capacity of microorganisms to withstand stressors such as high salinity and harmful substances, which enhances their overall performance [11,12]. The generation of reactive species by certain nanobubbles has the ability to alter microbial communities and accelerate degradation pathways [10]. Tests of NBs in two-stage AD systems revealed increases in yields of both hydrogen and methane, as well as improved acidity and hydrolysis [11,12].

However, to date, the NB research efforts in the AD sector, as summarised in refs. [10,11,12], has been based on mechanically generated NBs—which have drawbacks compared to the novel, electric-field-based approach described in the present study. Some key features of the highly novel and exciting electric-field nanobubble-generation approach for biogas upgrade are as follows:

- very energy-efficient;
- able to retrofit and increase the capacity of the existing footprint-constrained plant;
- readily amenable to scale-up and continuous-flow operation;
- portable, with easy shipping of nanobubble-generation equipment (e.g., skid-mounted);
- additive-free;
- removal of H_2_S in our single-step—nanobubble-formation process is also a unique approach;
- low methane slippage (~1.3 v%);
- no obvious regulatory difficulties.

These features distinguish this approach [13] quite dramatically from what is currently commercialised on the market, or even available in concept from fundamental knowledge of gas–liquid phenomena. In particular, the ability of the current NB-centric approach as applied to enhanced “water washing” as a CO_2_- (and H_2_S) removal technology is inherently more scalable than other CO_2_-stripping (or “biogas-upgrade”) approaches, owing, *inter alia*, to the lack of any need for a membrane or elevated pressures—meaning that gas compressor operations are not particularly needed for exit-stream pressure management emanating from the AD reactor itself. Added to these competitive NB-enhanced “water-washing” operations is the lower operating and capital costs of such membrane-free, lower-pressure operation—with the electric-field *modus operandi* obviating the need for moving parts and much in the way of maintenance (owing to no biofouling of non-existent membranes, as is sadly not the case with corrosion-prone impeller blades failing in compressors due to H_2_S attack, and fouled membranes of the status quo). The fact that essentially arbitrarily low or high capacity of biogas upgrade can be obtained means both upward and downward scalability in terms of biogas upgrade capacity—i.e., dramatically above and below the typical biomethane purification levels of 200–500 m^3^/h typically used in current biogas-stripping operations.

The overall goal of this study is to consider NB-generation applications in the AD sector—not just enhanced water washing *per se* to absorb preferentially CO_2_, but also for the attendant and important up- and down-stream water/sludge-treatment processes.

## 2. Methodology

Prior to discussing the results of the electric-field approach for generating NBs, it is perhaps useful to consider other approaches for NB generation and compare them to the electric-field method.

In the “porous-membrane” approach to generating nanobubbles, fluid flow through porous media is the foundation of the first primary mechanical NB-generation technique [14,15,16,17]. In order to create bubbles, particularly in water, pressurised injection gas is introduced through the medium membrane. Metastable bubble sizes of less than 1 μm can be attained by shrinking the medium’s pore sizes. Various nanobubble-generator companies, for instance, all employ systems that involve injecting a pressurised gas into a passageway followed by a fine-carbon-based porous medium with pore diameters ranging from several nm to several tens of nm. The porous medium should adhere to a specified shortest-to-longest distance ratio between the medium and the flow passage, whether it is oriented horizontally or tilted downward. Other crucial elements in this system are the gas pressure and fluid flowrate. Some suggest passing the liquid–gas mixture through a filter or porous medium to reduce the size of the bubbles and only allow bubbles smaller than a threshold to pass through, although the majority of methods involve injecting the gas through a fine porous medium to create nanobubbles. Sang, for example, is providing a system that draws raw water through a Venturi section and uses water flow to generate a relative pressure that permits the drawing of gas [18]. The gas–water mixture undergoes three filtering stages following pressurisation. Using a sponge, non-woven filter, or ceramic porous material, large bubble sizes and foreign particles are filtered in the first step. The second stage, which has beads stacked on top of one another, produces finer bubbles. The third stage, which has the same structural design as the first stage, filters the bubbles to create bubbles with almost the same size and nanoscale order.

In the “pressure-difference” category for generating NBs, a fluid may flow as a result of a mechanical crack between two points; the amount of turbidity in the flow depends on the crack’s size and other fluid characteristics. One of the basic principles utilised in certain techniques for the creation of nanobubbles is the mechanical pressure differential between two points in the flow of a liquid–gas mixture. In order to pressurise the gas–water mixture inside a tank, Toshihiko developed a pump that draws in gas and water [19]. The pressurised mixture is connected to the site of nanobubble production via a pressure-regulating valve, which regulates the mixture’s pressure at the generating location. The discharge section of the system has an orifice that helps control the pressure at which nanobubbles are produced. For this system to generate nanobubbles of the proper size and population, the ratio of P2/P1, where P2 is the pressure before the orifice and P1 is the mixture pressure before the pressure-regulating valve, must be controlled. Static mixers can also be used sparingly to create pressure differences. The cavitation principle of nanobubble generation—featuring in a number of industrial-generator designs—also exploits the creation of pressure difference, and thence nanoscale bubbles [17].

In the “shear-stress” method, a commonly used technique uses friction forces and shear stress to cause bubbles to gradually break into smaller ones until they are fine- and nano-sized. A few submersible NB-generator designs also use flow-rotational techniques, which are based on these frictional forces. Ho and others showed how a gas–water mixture passes through a chamber with a revolving shaft and various protruding units [20,21]. These units have the ability to rub the gas–water mixture and its surrounding surface, which could lead to smaller bubbles. The system is composed of several stages that first create microbubbles, which are subsequently reduced to create nanobubbles. Boundary-layer mixing is an optional feature that can be achieved by running a gas–water mixture through a lengthy hose. “Hammermill-rotation” and other submersible NB generators have a design with a “rotating-shaft” (and a porous membrane for bubble-size reduction towards the nanoscale range) [22] both use the general idea of rotational-swirl flow arrangements based on the idea of shear-stress/friction-induced progressive bubble-size reduction.

Nanobubbles and/or nanodroplets can be created in gas–water systems by applying an external static electric field [13] and exploiting dipole-orientation effects [5]. It is possible to create nanobubbles or nanodroplets by electrostrictively capturing a subpopulation of nanobubbles (or -droplets) from the outer reaches of the upstream/incipient population of macro-, meso-, or microbubbles when this external electric field is present close to a volume of liquid containing a medium. This external-field-induced method works well for converting a significant percentage of upward-rising macro- and/or mesobubbles, as well as bubbles that flow vertically or horizontally, into ultra-dense, long-lived NBs. This low-maintenance, solar-powered, energy-efficient “sheathed-electrode” approach, which lacks moving parts, is ideal for off-grid operations [13].

**Figure 1:**
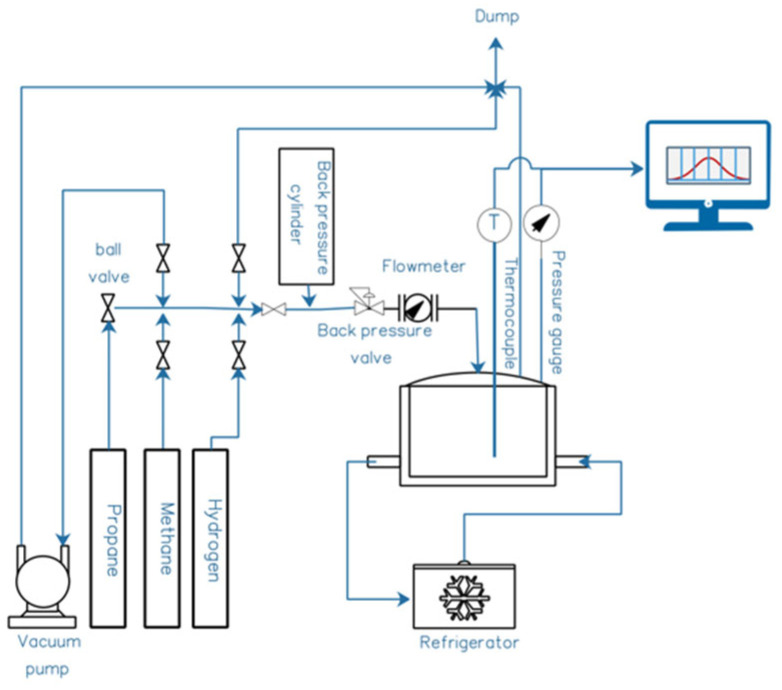
Schematic of gas–liquid pressure-vessel rig. The four main sections are Gas Supplier, Distribution Terminal, Reactor, and Refrigerator. During a NB-formation experiment, the reactor’s inlet valve is closed upon reaching the desired pressure, and pressure is logged digitally every second for the experiment’s duration, showing gas uptake into the liquid.

The novel method of NB formation was developed using an existing, state-of-the-art pressure-vessel rig—composed of a 0.3 L, 200 bar-rated stainless-steel pressure vessel linked up to a refrigerated temperature control system, with mechanical agitation on a rocking device. The details of the reactor are illustrated below in Figure 1.

In short, one purifes biogas by generating NBs using this additive-free and energy-efficient patented technology [7]. This is performed with the biogas in contact with liquid water (whether batch, continuous mode, fed batch, etc.). The reason for differential take-up of gas in nanobubble (NB) form into the water is that, even though de facto methane solubility is around 30 times higher than its Henry’s Law level in nanobubble form, this is still very small in terms of mass per volume in water (mg/L) in absolute terms. With conventional (standard molecular solvation) Henry’s Law solubility levels of CO_2_ and H_2_S’s being at least 40–50 times higher than CH_4_ in terms of absolute mass-per-volume in water (i.e., in mg/L), the increase in CO_2_ solubility due to NB creation though this breakthrough method results in much higher absolute f CO_2_ and H_2_S uptake into water. Quantitatively, mass-balance considerations show that CO_2_ and H_2_S levels transferred from the raw-biogas phase to the liquid (including in NB form) are approximately 16: and 25:1 compared to methane slippage into the water. So, we significantly purify methane, achieving ~95%-purity methane in a single-pass design). Microbes convert corrosive H_2_S, precipitating sulphur, with iron addition, to useful FeSO_4_.

At present, in proof-of-concept trials in a sub-litre pressure-vessel system (cf. Figure 1), enrichment of 60:40% *v*/*v* methane–CO_2_-biogas-mimicking gas mixture was up to ~96% *v*/*v* CH_4_.

**Figure 2:**
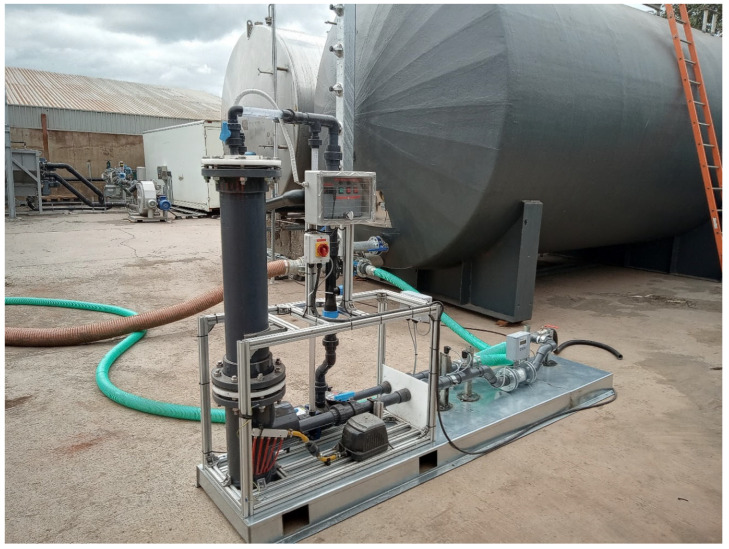
Photograph of NB-generation unit. The vertical cylinder is the electrostriction chamber.

Following laboratory proof-of-concept validation, a circa 120–180 L/min water flow was put through a sidestream on a continuous-flow AquaB (Naas, Ireland) wastewater-processing nanobubble-generation unit at a biogas/AD plant—albeit using municipal water as the CO_2_-capture agent. Gas flow was about 80 L/min at 6 bar g to a gas delivery manifold at the bottom of the electrostriction chamber using a compressor—in other words, a “driving force” of about 4 bar above the circa 2 bar g operating pressure of the unit. There is vortex-enhanced electrostriction, from upstream Venturi-screw microbubble generation from 40 L/min of atmospheric air drawn therethrough, and this converts the “mother” microbubbles into a sub-population of nanobubbles. This may be achieved by various gases. The already mentioned compressed gas supplied through a manifold connection at the bottom of the electrostriction column went upwards as a countercurrent gas supply line of generated microbubbles—followed by their conversion to nanobubbles on the internal electrode bank (cf. Figure 2). The power draw of the electrostriction itself from mains AC is only about 8 W, and the bulk of that is AC-to-DC conversion overhead. Additional power is needed for pumping and air compressors, although this is very low indeed considering the impressive levels of aeration that may be achieved very rapidly (cf. Section 3)—certainly a good deal less than a kiloWatt—so we may reasonably neglect much of the pumping costs in power-draw budgets. Although, to be fair, we perhaps allow a typical, industry guideline Venturi-draw power equivalence allowance of about 100–150 W. The compressor power was about 200 W up to ~1.5–2 bar g pressure inside the nanobubble generator.

The gases used were raw biogas, CO_2_, H_2,_ and air for three different sets of runs. Multiple passes on (waste)water were performed with ~2 and 8 wt% non-dissolved solids in the case of air NBs for post-processing digestate wastewater aiming to oxygenate it with air NBs, e.g., for oxidation of H_2_S, nitrite, and ammonia, and other odorous and highly reactive forms of sulphur and nitrogen—thus affording this post-AD wastewater important treatment (thus removing more toxic elements from residual sludge to make it more useful as an organic fertiliser). Another goal was to impart either CO_2_ or H_2_ NBs into thick post-pyrolysis water/sludge mixture (with a solids content of the order of ~35–40%) to be fed into the AD system, with the goal of boosting the yield of methane and enriching the methane content of the biogas above the typical level of ~60% *v*/*v*. Finally, municipal water was passed a number of times through the NB generator in contact with raw biogas being supplied, with about 60% *v*/*v* methane content, in an effort to remove preferentially CO_2_ and H_2_S in the form of NBs, whilst minimising methane slippage.

The incoming raw biogas was roughly 61 ± 1.5% wt. methane, with about 0.08% wt. H_2_S and the remainder as CO_2_. The temperature range of operation was 17 to 23 deg C, with an upstream pressure of 6 bar g and at 80 L/min for the gas flow supply. In terms of the internals of the NB generator pictured in Figure 1, the vertical section on the left housed the internal sheathed electrodes with 300 V DC applied, while a flow pattern was established across the bank of electrodes from vertically downward liquid flow pumped in from above this vertical section, from the right, as shown in Figure 1. Inside the vertical electrostriction section, the densification of the liquid (which entrains macrobubbles, with such bubbles “fed” upstream by a Venturi section) took place, and this allowed the fragmentation of this original population of macrobubbles into smaller nanobubbles when in contact with the electric field of the electrodes. In short, the densification of the water around the larger upstream bubbles mediated by the static field leads to a partial vacuum around these bubbles, which serves to suck fluid “pockets” of gas into a population of “satellite” nanoscale bubbles surrounding the periphery of the original macro-/mesobubbles.

**Figure 3:**
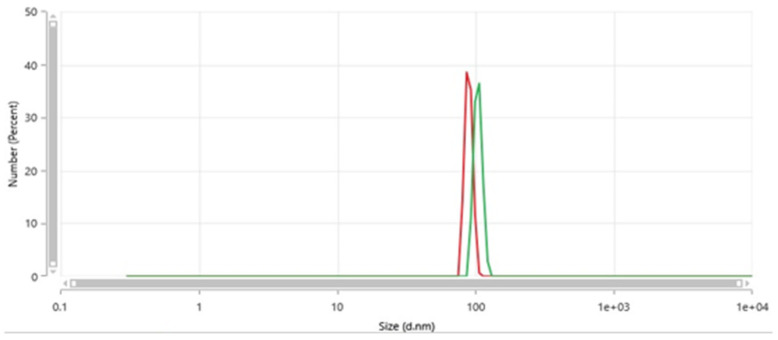
A couple of typical size distributions from Dynamic Light Scattering of filtered water passed through the nanobubble generator showing evidence of nannobubbles (absent in the water source).

Figure 3 shows a couple of typical size distributions from Dynamic Light Scattering via a Malvern Zetasizer Pro (Malvern, UK) of filtered water passed through the nanobubble generator (such that the derived count rate is less than 100 k.c.p.s. [13]); in any event, this water source shows no such nanoscale light-scattering features prior to passage therethrough (i.e., well below any instrument detection limits). In these examples, the typical diameter range is around 85–105 nm. The scale is up to 10 microns, and only these nanobubble features are present (with no detectable level of microbubbles); as such, light-scattering does not detect larger meso- and macrobubbles.

It should be noted that the industrial equipment produces NBs in this size range—although the electric-field approach, in general, tends to lead to NB sizes in this typical range, which are favourably lower compared to other methods [13].

## 3. Results and Discussion

### 3.1. Raw-Biogas Upgrade

For biogas upgrade, about 93.6% purity was achieved on a single-pass basis, rising to 96.1% for double-pass recirculation of municipal water—all performed at about 1.5–2 bar g. The energy cost was about 0.048–0.095 kWhr/Nm^3^ (depending on single- or double-pass flow, with single-pass preferred for smaller membrane systems). This is a good deal lower in comparison to amine scrubbing (typically in the broad range of 0.1 to 0.5 kWhr/Nm^3^, or more [23]), and the narrower range of 0.17–0.24 kWhr/Nm^3^ for membrane systems [24]. Multi-angle Dynamic Light Scattering (DLS) measurements indicated NB populations of ~1–2 × 10^8^ NBs per ml, while off-gas analysis revealed that the gas in the NBs was about 98.8% *v*/*v* CO_2_ [13]. The methane “slippage” from the gas stream was about 1.35 ± 0.08% vol.—which is not unreasonable, although falling slightly short of the 1% target that most biomethane operators would set as a final value. This level of methane slippage arises as an inevitable byproduct of electrostrictive gas uptake into nanoscale pockets, or packets, of gas forming (or nucleating) at the periphery of macrobubbles, i.e., the temporary vacuum surrounding macrobubbles by a retreating interface born of electrostriction does attract, statistically, a certain amount of methane—albeit comparatively little in comparison to the quadrupolar CO_2_ molecules (and their significant quadrupole–dipole coupling in water).

So, given the low energy and operational, low-pressure ease of operation, some level of methane slippage may well be operationally and economically acceptable. Still, increasing water flowrate slightly for a higher ratio of water washing to raw biogas is a simple expedient that would probably improve this straightforwardly—and the gas/liquid flowrates can be optimised in a broader scan of Lockhart–Martinelli two-phase-flow parameter space.

### 3.2. AD Feed Sludge Water Nano-Carbonation and -Hydrogenation

In terms of the CO_2_ and H_2_ feed upstream to post-pyrolysis sludge upstream of AD, both CO_2_ and H_2_ addition in the form of NBs led to enhanced biomethane yield over 30-day laboratory AD-culturing, when using this “nanobubby sludge” as an AD feedstock. Here, the cylinders and biogas feeds were linked to the Venturi section, and the sludge-water flow was recirculated up to several times. The pressure in the electrostriction section was about 1.5 bar g. So, the pressure difference “driving force” for CO_2_ and H_2_ injection (in the form of NBs) was about 1 bar g. There were three passes of 1000 L of IBC-borne post-pyrolysis sludge water put through the unit (from and to IBCs), each time with a similar water and H_2_/CO_2_ flowrate. Earlier, high-pressure tests in the sub-lite pressure-vessel rig at about 70–80 bar g for CO_2_-NB generation showed a drop in pH down to about 4.3 (measured after about two days following post-experiment exposure to atmosphere), owing to the higher level of solubility from Henry’s Law with the high pressure of CO_2_ driving this (with NBs providing about 45–50% extra CO_2_ mass). For lower-pressure generation of NBs (around 1–2 bar g) in the larger-scale continuous-flow system, the initial drop in pH was not far behind these earlier results, at about 4.7. It was found that the pH of the control sludge was 6.9, whilst that of the first, second and third pass were initially about 4.6, 4.45, and 4.35, respectively.

It was found that the AD biomethane yield was boosted by *circa* 6 and 5%, respectively, by adding CO_2_ and H_2_ NBs in the flow-based NB generator to the post-pyrolysis AD feed sludge water. This is an important finding at process plant, industrial level.

### 3.3. Downstream Water-Sludge Treatment

In the case of digestate-water treatment using air nanobubbles applied to both the ~2 and 8 wt% solids post-AD sludge water streams (of much interest for reuse of detoxified and dewatered residual sludge as organic-fertiliser feedstock [1,6]), these are revealed in Table 1a,b, including both Biological Oxygen Demand (BOD) and Chemical Oxygen Demand (COD). The values of initial samples are compared for passage without generating NBs, i.e., with the electric-field action disabled—it is clear that neither of these “control” cases is equivalent to the case of NB generation. Compared to the initial samples, there were good conversions to nitrate from, *inter alia* nitrite, owing to reactive oxygen species (ROS)—cf. Table 1. Over multiple passes, nitrite levels fell, e.g., in the 8 wt% whole-digestate case from 31.6 mg/L (N) to 12.8 mg/L (N) after the third pass; there is some, less spectacular, drop after a single pass, which points to the reorganisation of the NBs from a virgin state to a revised population in terms of ROS activity for oxidation chemistry (i.e., in going to a second pass instead for the first instance of non-virgin nanobubbles). Nitrite conversion results were, encouragingly, even more robust for the 2%-digestate case, given that there was a larger NB population per unit digestate mass. The ammonia level was partly reduced after one pass but dropped substantially after the second and third pass—also consistent with reorganisation of non-virgin NBs (and the associated boost in ROS populations from that process), as determined by NB-population analysis by multi-angle DLS [13]—also, cf. Figure 3. Although it was not possible to measure ROS directly, it was found that the measurement of the shift in Oxidative Reductive Potential (ORP) led to a rough proxy measure for the level. In that sense, it was estimated that ROS population increases from reorganisation of non-virgin NBs may have been roughly 1.5 times greater than those of virgin-NB generation (or NB nucleation). Similarly, conversion to nitrate improved in going from the first to the second/third pass—pointing to rises in ROS activity by reorganisation non-virgin NBs—effectively, passage through the NB generator on subsequent passes helps to break up some of the “cloaks” of adsorbed species enveloping the NBs, which leads them to boost the ROS population and the accompanying oxidative activity. This is seen explicitly by the level of oxidised N increasing from 2.5 to 11.4 mg/L over three passes (for the 8% wt solids whole-digestate case), with the major “step-change” on foot of the second pass (i.e., the first occasion that non-virgin NB “colonies” and their electrostatically adsorbed colonies are disrupted). Similar trends are seen in the oxidation of H_2_S and phosphorous species—and especially so for the 8% wt solids whole-digestate case.

It can also be seen that there is a somewhat greater proportional effect in terms of affecting oxidative-driven changes by NB aeration in the case of the ~2% compared to the ~8% wt aqueous digestate-sludge stream: there is a greater concentration of reactive oxygen species in the former case vis-à-vis species of nitrogen, phosphorous, and sulphur in highly reactive form, in terms of proportionate oxidative capacity of the NB-aerated sludge water. Still, in the case of the 8% wt digestate stream, the same qualitative trends are clearly visible, in that the active oxidation of the digestate water takes place in a highly effective manner (cf. Table 1b vs. Table 1a).

For a single-pass operation, the nitrite conversion to nitrate is rather spectacular (albeit with other nitrogen sources also playing a part), whilst ammonia oxidation (despite influent levels not being problematic) is also notable—all of which are seen in the substantial increase in total oxidsed nitrogen. BOD and COD reduction is also meaningful and important.

As can be seen in Table 1, there were improvements in ammonia, nitrate, nitrite, BOD, and COD, and %-change data is instructive. The largest improvements were seen in BOD, COD, and nitrite-to-nitrate conversion upon primary aeration on the first pass—in the very creation of virgin nanobubbles in the first instance. However, the more subtle effects of reactive oxygen species (ROS) tend to be apparent with the subsequent passes in oxidation of ammonia, in dealing with non-virgin NBs. The same trends were also seen for highly reactive phosphorous and sulphur in the guise of oxidation of these species, with attendant improvements in odour (although this latter observation is, admittedly, more anecdotal).

From an operational perspective, the number of passes should be determined based on the desired purity level of key target parameters, while considering the energy cost of recirculation for multi-pass operations. 

## 4. Conclusions

The present study has witnessed exciting applications of nanobubble gasification for various important applications in the anaerobic-digestion sector. The energy for biogas upgrade was about 0.48–0.95 kWhr per Nm^3^ on a single-pass basis, with up to 95–96% purity and about 1.35% methane slippage—making this an ideal, lower-cost/lower-energy preliminary-treatment option—effectively, a superior water-washing technology. This can be deployed prior to more effective “latter-stage” treatment by membrane technologies for CO_2_ (and H_2_S) removal. The highly scalable version of this NB-empowered “water-washing” technology makes it highly economic at lower throughput levels and flowrates of AD-based biogas production [9], although it can be deployed to substantially higher levels of biomethane production as well. Working hand in glove as an effective pre-treatment step ahead of less intensive downstream membrane treatment (if Grid-injection or food/beverage methane-purity levels are desired), this approach reduces both the capital and operating costs of membrane treatments, with a much lower downstream membrane treatment capacity needed—also prolonging the useful operating life of biogas upgrade membranes with less intensive maintenance schedules needed. Given that this differential—and, indeed, preferential—CO_2_ and H_2_S capture takes place essentially at ambient pressure, e.g., of the order of 0.5–1.5 bar g (as opposed to ~12–18 bar g in conventional amine scrubbing or membrane capture approaches to remove CO_2_), then very little (to no) gas compressor capacity is needed—which reduces both operational and capital cost, and associated maintenance schedules, a very great deal; indeed, this is especially important, given that H_2_S poisoning of compressor blades upstream of raw-biogas upgrade itself is an often-disabling and crippling problem in industrial biogas production, which is often ignored and afforded too little engineering operational attention in comparative technological analyses [1]. However, if simple methane purity levels of the order of 94–95% are desired for gas engine and gas turbine usage, then the present NB-enabled water-washing technology may be contemplated directly, without the need for downstream membrane installation, and with little threat of H_2_S poisoning to gas turbine/engine operation (e.g., minimisation of sulphide-related corrosion attack after the inlet-manifold section or in the combustion cylinders themselves).

The prospect of a simple course of nano-carbonation or –hydrogenation upstream of AD itself, in the post-pyrolysis sludge water stream, is also of much interest, and is operationally relatively simple. The finding of improved biomethane yield is encouraging, as the excellent surface-area-to-volume ratio afforded by the nanobubbles allows for a greater thermodynamic incentive towards biomethane production reactions in the AD chamber to push more towards the product: the boost in mass-transfer coefficient of the CO_2_ and H_2_ allows for Fick’s Law-driven diffusion of these from NB form into a higher regularly solvated liquid-phase concentration to “push” AD reactions more towards thermodynamic completion [1,9]. If anything, this may be an even more operationally facile and nearer-term scope for the deployment of NB technology for water treatment in the renewable-gas sector.

Turning to nano-aeration (or even oxygenation) of the downstream digestate sludge-water in that substantial water treatment challenge (with opportunities for prime organic-fertiliser production), there are substantial gains to be made from low-pressure aeration of this water stream, with the remarkable oxidative capacity of air nanobubbles on display at lower capital and operating expense. This is an advance similar in performance to Advanced Oxidation Processes at far lower operating cost, and with membrane-free and low-maintenance operation [13].

Taken together, the findings of the present study indicate ample scope for electric-field nanobubbles technology to make substantial contributions to water treatment in the renewable-gas sector. However, although the present study acts as an important “proof-of-concept”, one also needs to consider the challenges of the wider industrialisation of this process. Although the operational cost in applying nanobubbles for “water washing” applied to biogas upgrade is very competitive (with beneficial H_2_S removal in the water also for lowering corrosive damage to compressor blades), as well as the low energy cost for downstream, post-AD digestate-water treatment, it must also considered that there are other operational and process barriers to be overcome in advance of widespread renewable-gas-industry uptake of this innovative technology. The main among them are the following: (i) the need for larger water flow in biogas upgrade *per se*, together with make-up and recycle-line engineering, (ii) feedforward ratio control loops for dissolved CO_2_ levels in the make-up and recycle water-flow lines, (iii) implementation of strict DC-voltage electrical safety standards with CE- and UL-certification (or equivalent), and (iv) IP- and ATEX ratings for efficient outdoor and all-weather usage, as well as (v) ISO Environmental Technology certification. In addition to these engineering and certification standards, including for operational control, the near-real-time measurement of water quality from downstream sludge treatment needs to be considered for incorporation into a feedback control loop for digestate-water flow, as well as potential voltage and DC duty-cycle variation using Model-Based Control strategies. Naturally, these control- and quality-engineering strategies are complex and involved and offer challenges to the scale-up of this technology, despite the prognosis for scalability being quite positive.

## Figures and Tables

**Table 1:** (**a**): Highlights for 2 wt% digestate; % change is relative to the initial sample, except for pH, where the absolute change is indicated. (**b**): Highlights for 8 wt% digestate; % change is relative to the initial sample, except for pH, where the absolute change is indicated.

mg/L Below	Initial Sample	Passage Without NB Generation	1st Pass	2nd Pass	3rd Pass
(**a**)					
Ammonia	58.5 ± 0.7	56.2 ± 0.8	34.6 ± 0.9 (−41)	27.3 ± 0.8 (−53)	21.9 ± 0.7 (−63)
BOD, mg/L	734 ± 11	680 ± 12	353 ± 13 (−52)	257 ± 14 (−65)	192 ± 11 (−74)
COD, mg/L	952 ± 26	870 ± 21	452 ± 19 (−52)	364 ± 17 (−62)	289 ± 13 (−70)
Nitrite, mg/L	16.7 ± 0.31	15.3 ± 0.22	5.02 ± 0.14 (−70)	3.64 ± 0.12 (−78)	2.94 ± 0.11 (−82)
Nitrate, mg/L	1.54 ± 0.07	1.68 ± 0.06	6.22 ± 0.23 (304)	8.63 ± 0.26 (292)	10.3 ± 0.32 (356)
pH	7.81 ± 0.06	7.73 ± 0.04	7.32 ± 0.05 (−0.49)	7.04 ± 0.04 (−0.77)	6.94 ± 0.03 (−0.87)
Total nitrogen, mg/L	89.4 ± 1.9	82.4 ± 1.7	56.6 ± 1.4 (−37)	49.0 ± 1.2 (−45)	47.2 ± 1.0 (−47)
Total oxidised nitrogen, mg/L	0.91 ± 0.02	0.97 ± 0.02	1.52 ± 0.05 (69)	3.37 ± 0.17 (275)	3.74 ± 0.19 (316)
H_2_S, mg/L	31.4 ± 0.6	29.2 ± 0.5	18.9 ± 0.5 (−40)	14.7 ± 0.4 (−53)	11.3 ± 0.4 (−64)
Phosphorus, mg/L	28.7 ± 0.7	26.3 ± 0.8	18.6 ± 0.7 (−35)	14.8 ± 0.5 (−48)	11.9 ± 0.6 (−59)
(**b**)					
Ammonia	117 ± 4	109 ± 4	77.0 ± 2.4 (−34)	58.0 ± 2.1 (−50)	48.0 ± 2.2 (−59)
BOD, mg/L	1120 ± 43	1030 ± 41	568 ± 22 (−49)	453 ± 17 (−59)	390 ± 16 (−65)
COD, mg/L	1280 ± 28	1180 ± 25	783 ± 20 (−39)	523 ± 18 (−59)	452 ± 17 (−65)
Nitrite, mg/L	31.6 ± 1.2	29.6 ± 1.1	15.4 ± 0.8 (−51)	13.5 ± 0.7 (−57)	12.8 ± 0.8 (−59)
Nitrate, mg/L	2.13 ± 0.16	2.37 ± 0.18	11.2 ± 0.53 (426)	15.0 ± 0.61 (604)	16.1 ± 0.63 (656)
pH	7.92 ± 0.05	7.81 ± 0.04	7.52 ± 0.04 (−0.4)	7.29 ± 0.03 (−0.63)	7.16 ± 0.05 (−0.76)
Total nitrogen, mg/L	179 ± 11	169 ± 10	142 ± 9 (−21)	123 ± 6 (−31)	116 ± 5 (−35)
Total oxidised nitrogen, mg/L	2.5 ± 0.2	3.2 ± 0.3	13.6 ± 0.9 (224)	19.6 ± 1.1 (292)	21.4 ± 1.0 (356)
H_2_S, mg/L	51.2 ± 2.1	48.7 ± 1.7	36.7 ± 1.6 (−28)	31.3 ± 1.6 (−38)	26.4 ± 1.5 (−48)
P. mg/L	41.3 ± 1.3	39.2 ± 1.1	29.4 ± 0.9 (−29)	22.4 ± 0.8 (−46)	19.7 ± 0.7 (−52)

## Data Availability

Data is contained within the article.
